# The role of programmed cell death in osteosarcoma: From pathogenesis to therapy

**DOI:** 10.1002/cam4.7303

**Published:** 2024-05-27

**Authors:** Suqing Liu, Chengtao Liu, Yian Wang, Jiewen Chen, Yujin He, Kaibo Hu, Ting Li, Junmei Yang, Jie Peng, Liang Hao

**Affiliations:** ^1^ Department of Orthopedics, The Second Affiliated Hospital, Jiangxi Medical College Nanchang University Nanchang China; ^2^ Queen Marry College Nanchang University Nanchang China; ^3^ Shandong Wendeng Osteopathic Hospital Weihai China; ^4^ The Second Clinical Medical College, Jiangxi Medical College Nanchang University Nanchang China; ^5^ Department of Sports Medicine, Huashan Hospital Fudan University Shanghai China

**Keywords:** osteosarcoma, pathogenesis, programmed cell death, therapy

## Abstract

Osteosarcoma (OS) is a prevalent bone solid malignancy that primarily affects adolescents, particularly boys aged 14–19. This aggressive form of cancer often leads to deadly lung cancer due to its high migration ability. Experimental evidence suggests that programmed cell death (PCD) plays a crucial role in the development of osteosarcoma. Various forms of PCD, including apoptosis, ferroptosis, autophagy, necroptosis, and pyroptosis, contribute significantly to the progression of osteosarcoma. Additionally, different signaling pathways such as STAT3/c‐Myc signal pathway, JNK signl pathway, PI3k/AKT/mTOR signal pathway, WNT/β‐catenin signal pathway, and RhoA signal pathway can influence the development of osteosarcoma by regulating PCD in osteosarcoma cell. Therefore, targeting PCD and the associated signaling pathways could offer a promising therapeutic approach for treating osteosarcoma.

## 1 INTRODUCTION

Osteosarcoma is a primary solid malignancy especially occur in bone and rarely in the soft tissue such as skin, ligament, and muscle. It is derived from the mesenchymal cells (MSCs).^1^ This sarcoma is most easily acquired by adolescents between 10 and 25 years old, and the proportion of adolescent patients is about 70% in all osteosarcoma patients. In addition, it also affects people about 60–75 years old.^2^ Osteosarcoma has a high rate of metastasis and recurrence, making it a difficult sarcoma to manage and difficult to treat with a low clinical survival rate. The metastasis rate of osteosarcoma is more than 85%.^3^, ^4^ The occurrence location of osteosarcoma is relatively random. It can originate from the metaphyseal portion of any bone, but the femur and tibia are more common sites. Additionally, the knee joint is the most common site for osteosarcoma, accounting for over 50% of all cases,^5^, ^6^, ^7^, ^8^ and in later stages, osteosarcoma is highly migratory and can migrate to either the closely site of primary site or distant organs such as lung, cerebral tissue, and liver. In addition, the lung parenchyma is the most common site for metastasis. The symptoms of osteosarcoma are some common symptom, mainly local pain, swelling, and spontaneous fractures, but the symptoms worsen over time and progress to intolerable pain that it resistant to painkillers. As the cancer cells metastasize and affect other organs, systemic deterioration such as weight loss, anemia, and cachexia will occur. The diagnosis of osteosarcoma can be diagnosed by X‐ray, CT, etc., but biopsy is the most important diagnostic method.^9^, ^10^

The pathophysiology mechanism of osteosarcoma is complexed, but the rapid bone growth during adolescents has a deep relationship with the osteosarcoma. In addition, a small number of osteosarcomas are caused by radiation exposure, exposure to alkylating agents and genetic mutation.^1^, ^11^ Many recent experiments and articles on the mechanisms of osteosarcoma production have found that programmed cell death (PCD), such as apoptosis, pyroptosis, necroptosis, ferroptosis, autophagy, and cuproptosis, is also important to produce osteosarcoma.^4^, ^12^, ^13^

The mode of cell death can be separated into two parts: the accidental cell death and PCD.^14^ PCD is a kind of cell death which is regulated by many genes and factors, such as PI3K/AKT signal pathway, Wnt/nt/way, and PI3K/AKT signal paRhoA signal pathway. Its effect on the human body is twofold; it can contribute to the normal functioning of the organism, and at the same time, it can cause damage to the organism.^15^ Various forms of programmed cell death (PCD) exert distinct roles in osteosarcoma development. In the context of apoptosis, the majority of osteosarcoma patients exhibit dysfunctional apoptotic pathways, disrupting the balance between cell proliferation and death. This imbalance contributes to tumor initiation and metastasis.^16^ Pyroptosis is an inflammatory cell death mediated by Gasdermin (GSDM) and has an important role in preventing tumor production. However, evidence suggests that pyroptosis have a dual role in osteosarcoma development. It can both promote the onset of osteosarcoma and inhibit its progression, depending on different signaling pathways.^17^, ^18^ Ferroptosis is a non‐conventional type of PCD that results from the accumulation of ROS and is iron‐dependent,^19^ and it has been clinically demonstrated that a variety of ferroptosis inducers such as RSL3 and erastin can promote the production of ferroptosis and thereby destroy tumor cells.^20^, ^21^, ^22^, ^23^ Autophagy is a process by which cells degrade damaged structures and reuse the broken‐down material.^24^ Autophagy is closely related to osteosarcoma. Autophagy can resist chemotherapy and promote cell proliferation and survival, so the regulation of autophagy can also be considered as a new target for the treatment of osteosarcoma. In addition, autophagy also has dual role to osteosarcoma; it can both promote and inhibit the development of osteosarcoma depending on different mechanism.^25^, ^26^, ^27^, ^28^ Necroptosis is induced by TNFR1/RIP1, 3/MLKL signal pathway, which damage cell structure.^29^ The long noncoding RNA (lncRNA) has been proved to have high connection with osteosarcoma.^30^ The cuproptosis is a copper ion‐dependent cell death. Although his specific relationship to osteosarcoma has not been studied, numerous experiments have found a strong link between cuproptosis‐related genes or cuproptosis‐related lncRNA and osteosarcoma.

The therapeutic approaches for osteosarcoma are diverse, encompassing conventional methods like chemotherapy and radiotherapy, which are standard treatments in cancer therapy. However, in the 21st century, advancements in molecular technologies have led to the development of targeted drugs that focus on signaling pathways involved in programmed cell death (PCD). These targeted therapies offer promising avenues for treating osteosarcoma by specifically targeting the molecular mechanisms underlying tumor growth and progression. For example, arsenic trioxide (ATO) can activate the p38, JNK, and AMPK to induce apoptosis which can treat osteosarcoma. Proteasome inhibitor can also be used to increase ATO function.^31^ What's more, drug targeted on mitochondria can activate immunogenic pyroptosis to clear osteosarcoma.^32^ In addition, some immune checkpoint treatment can also be used.^33^ So, the PCD and its signal pathway can be a new target for osteosarcoma treatment.

## 2 MECHANISMS OF CELL DEATH IN OSTEOSARCOMA

Programmed cell death has been an active topic for decades and continues to be continually supplemented and improved. It is closely related to the development of osteosarcoma. Below, we will introduce the various roles of programmed cell death in osteosarcoma.

### 2.1 Apoptosis

Apoptosis is the main pathway of regulated cell death, and it is achieved through two apoptotic pathways: the extrinsic pathway and the intrinsic pathway, also known as cell death receptor pathway and mitochondrial apoptotic pathway, respectively.^34^

Extrinsic apoptosis can be brought on by substances that damage DNA, such as DOX, CIS, and ETOP.^35^ Additionally, this pathway requires death ligands to bind to their corresponding death receptors, triggering the assembly of the death‐inducing signaling complex (DISC). For instance, Fas or other death receptor‐associated death domain protein can interact with Caspase‐8 and Caspase‐10, subsequently activating them. This activation leads to the stimulation of executioner caspases including Caspase‐3/7/6.^34^

Numerous triggers, including oxidative stress and hypoxia, initiate the intrinsic apoptotic pathway.^36^ Intracellular stimuli can trigger the opening of the mitochondrial permeability transition pore (PTP), resulting in the rupture of the outer mitochondrial membrane (MOM), a process known as mitochondrial outer membrane permeabilization (MOMP). This event enables the release of various pro‐apoptotic proteins from the mitochondrial intermembrane space into the cytoplasm.^12^ Although the mechanism is not fully understood, current study confirms that MOMP causes the release of cytochrome c (CytC) to aid in the formation of apoptosome, which is then used for the activation of Caspase‐3 and Caspase‐7.^37^

This apoptosis pathway is regulated by a multitude of pro‐apoptotic and anti‐apoptotic proteins. Among them, the B‐cell lymphoma 2 (Bcl‐2) family is one of the proteins that is particularly important for apoptosis, comprising three subfamilies: anti‐apoptotic, pore‐forming proteins, and Bcl‐2 homology 3 (BH3)‐only proteins. While pore‐forming proteins and BH3‐only proteins exhibit pro‐apoptotic properties, anti‐apoptotic members, such as Bcl‐2, B‐cell lymphoma‐extra‐large (Bcl‐XL), B‐cell lymphoma‐w (Bcl‐W), and myeloid cell leukemia 1 (Mcl‐1), impede the formation and activation of the apoptosome.^12^ In summary, the activation of pro‐apoptotic protein and the inhibition of anti‐apoptotic protein promote apoptosis, contributing to our understanding of the mechanisms underlying osteosarcoma.^38^

**FIGURE 1:**
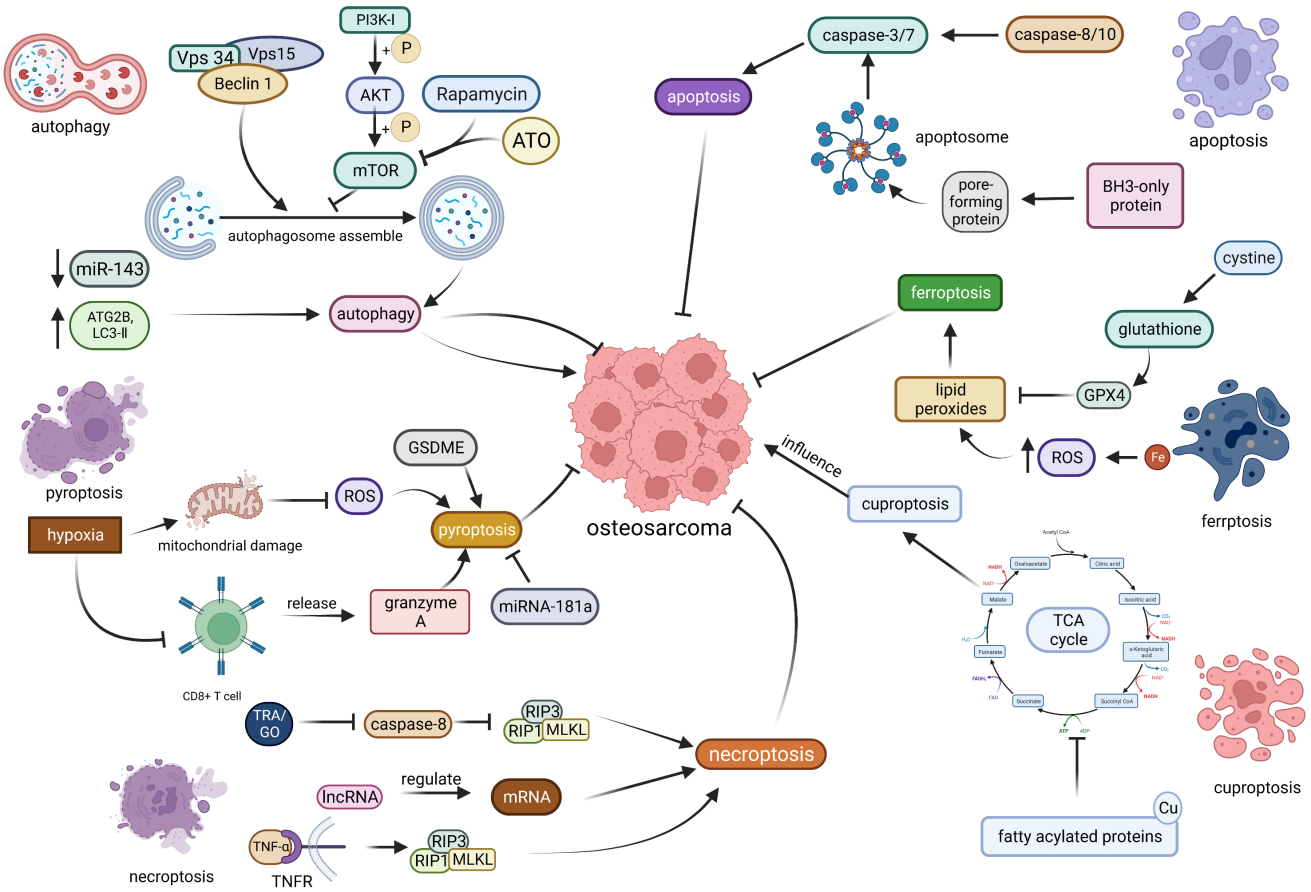
Core molecular mechanism of PCD in osteosarcoma. Vps34, a mammalian class III PI3K, forms a complex with Vps15 and Beclin 1 that contributes to autophagosome assembly. Phosphorylated PI3K‐1 phosphorylates AKT, which activates mTOR, which inhibits autophagosome assembly. Rapamycin and ATO (arsenic trioxide) reduce the inhibitory effect of mTOR and induce autophagy. The downregulation of miR‐143 and the upregulation of ATG2B and LC3‐II contribute to autophagy, which exhibits both inhibitory and promotive effects on tumorigenesis. Autophagy plays a dual role in tumor development, acting both as a suppressor and as a promoter. Pyroptosis is mainly suppressed by hypoxia, which reduces mitochondrial ROS production and consequently diminishes focal cell death generation. Hypoxia can also inhibit the function of T cells, and GSDME and granzyme A produced by T cells can promote the generation of pyroptosis. MiRNAs can inhibit pyroptosis. In necroptosis, TRA/GO can inhibit Caspase‐8, thereby contacting the inhibition of RIP3 complex by Caspase‐8 and promoting necroptosis. LncRNA and TNF‐a can also control necrotic apoptosis generation. Cuproptosis can influence the development of osteosarcoma but the detail function and mechanisms are unclear. This figure has been created at https://app.biorender.com (accessed on 16 July 2023).

In osteosarcoma, dysregulated expressions of Bcl‐2 family members have been discovered, which also means that apoptosis is a promising therapeutic target. According to the study, this dysregulation was correlated with the prognosis of osteosarcoma patients, with those exhibiting high levels of Bcl‐2 expression had a poorer prognosis and stronger tumor metastasis compared to patients with low levels of Bcl‐2 expression. Trieb's study showed that in all osteosarcoma research patients 46% patients express more Bcl‐2 than normal and 35% patient have a clearly overexpression level of Bcl‐2.^39^ Another study demonstrated that anti‐apoptotic Bcl‐XL was overexpressed in high metastatic osteosarcoma cell line (M8) in comparison with low metastatic osteosarcoma cell lines (Saos‐2, MG63, and U2OS), along with other anti‐apoptotic members.^40^ But surprisingly, only combined inhibition of Bcl‐XL and Mcl‐1 produced significantly higher antitumor effects, rather than in combination with Bcl‐2 inhibitors.^41^ Several novel treatments have surfaced in recent years. Mcl‐1 inhibitors have been developed and proven to be effective in many types of tumors when used alone or in conjunction with venetoclax, but it is still unknown if they are also effective in treating osteosarcoma. Venetoclax can inhibit the Bcl‐2 and disrupting the survival signals of cancer cell. Therefore, further research is necessary to successfully target the Bcl‐2 family for the treatment of osteosarcoma OS^42^ (Figure 1).

### 2.2 Ferroptosis

Ferroptosis is a novel type of PCD that differs from other cell death modalities in terms of morphological, biological, and genetic levels.^19^ It is driven by fatal lipid peroxidation that is iron‐dependent and brought on by abnormalities in cellular metabolism and redox homeostasis.^43^ The most important feature of this is an imbalance of lipid hydroperoxides and ferrous ion (Fe^2+^), which is caused by excessive reactive oxygen species (ROS), leading to oxidative stress.^44^ More and more studies have demonstrated that ferroptosis plays an important role in regulating the onset and progression of orthopedic diseases, including osteosarcoma. Therefore, figuring out the ferroptosis regulatory target may lead to new opportunities for treating osteosarcoma. For example, the traditional ferroptosis inducer such as RSL3 and ferrostatin‐1 can be used to treat some cancer.^23^

Unlimited lipid peroxidation is a sign of ferroptosis.^43^ The process initiates with the release of a bisallylic hydrogen atom from the polyunsaturated fatty acyl (PUFA) component of the phospholipid bilayer, generating a carbon‐centered radical. Subsequently, this radical reacts with oxygen to form a peroxy radical.^45^ Free‐radical‐mediated reactions have the potential to compromise the integrity of the cell membrane and expose the contents of the cell to the extracellular environment if the free radicals are not converted to lipid hydroperoxide and reduced to the matching alcohol in a timely manner.^46^ Cell death occurs in the end. As a result, membranes containing a high PUFA content are prone to peroxide, which means that ferroptosis is more likely to occur. In addition, meta‐analysis of the antitumor function of n‐3 PUFA has proved that the n‐3 PUFA administration can inhibit the function of IL‐6 and TNF, which can promote the tumor growth and metastasis. It is true that high dose of TNF can kill cancer but at the low level it will promote tumor development.^47^

GPX4‐regulated ferroptosis is a classical ferroptosis inhibition system. In osteosarcoma, high expression of GPX4 allows it to avoid ferroptosis, increasing its chances of surviving. In the presence of glutathione (GSH), GPX4 can reduce lipid peroxides to corresponding lipid alcohols, thereby negatively regulating ferroptosis.^48^, ^49^ Cysteine is imported into the cytosol via the System XC‐ (consisting of transmembrane protein complex SLC7A11 and SLC3A2) in this metabolic pathway, where it is converted into cysteine to create GSH.^50^ Therefore, depletion of either GPX4, System XC‐, and the GSH levels can result in ROS overproduction and accumulation of lipid peroxides, ultimately leading to ferroptosis.^51^ So, one of the ways to induce ferroptosis is to block the function of the System XC‐, which can be achieved by erastin. This also provides insights for the treatment of osteosarcoma. However, several innovative treatments have been developed as conventional adjuvant chemotherapy techniques have reached a bottleneck for osteosarcoma. Studies have shown that bavachin can downregulate the expression of SLC7A11 and GPX4 while upregulating the expression of transferrin receptors like p53 in osteosarcoma.^52^ Among these, p53 controls SLC7A11 upstream, which has an inhibiting effect. What's more, the tirapazamine (TPZ) can also downregulate the expression level of SLC7A11 and GPX4 and increase the level of MDA and Fe^2+^ which can lead to osteosarcoma cell produce more ROS inducing ferroptosis.^53^ Therefore, the combination of various effects can initiate tumor cell ferroptosis to treat osteosarcoma.

Although the understanding of the mechanism of ferroptosis has advanced significantly, it is still unclear how cells ultimately die. In summary, negative regulation of ferroptosis protects cells from the cytotoxic effects of lipid peroxides, while positive regulation of ferroptosis can be used to kill osteosarcoma cells. Thus, novel ferroptosis activators have become a hot research field in biology. In other words, further in‐depth study of the mechanism of ferroptosis and the trial of ferroptosis targeted drugs will help continued development of osteosarcoma treatments.

### 2.3 Autophagy

Autophagy is a normal PCD, which is activated at the situation when cells are under the stressful conditions, such as cellular disruption, oxidative stress, and chromosomes damage. It can induce decomposition of cell structure and reuse it to generate new cellular component.^54^ As caspase‐independent PCD, autophagy realize it function by lysosomes, so, the chromosome aggregation and organelle swelling seen in other kinds of PCDs does not occur in autophagy^55^ In terms of the relationship between autophagy and osteosarcoma, autophagy has a dual role. It can protect osteosarcoma cell and allow them to survive, but it can also inhibit the transformation of tumor cells or kill it through other kind PCD, which can inhibit tumor development and metastasis.^56^, ^57^ When tumor cells are deprived of nutrients and oxygen, autophagy promotes the breakdown of own broken and old structures, and then, the cell utilizes the decomposition, which can prevent apoptosis of tumor cells. Furthermore, in certain circumstances, autophagy can also facilitate the metastasis of osteosarcoma cells. So, the role of autophagy in osteosarcoma need to analysis individually.^58^, ^59^ Meanwhile, the effect of autophagy is different for different lineages of osteosarcoma cells, for example, gemcitabine (GCB)‐induced autophagy promotes cell death in the CCH‐OS‐D osteosarcoma cell line but inhibits cell death in the LM7 osteosarcoma cell line. The CCH‐OS‐D and LM7 cell lines are human osteosarcoma cell line.^60^ In osteosarcoma development, the PI3K/AKT/mTOR signaling pathway can inhibit autophagy. So, this signal pathway can be used as a target in the treatment of osteosarcoma. For example, rapamycin and ATO can decrease the inhibitory effect of PI3K/AKT/mTOR signaling pathway on autophagy for the treatment of osteosarcoma.^61^, ^62^ Some tumor suppressors such as RB1, TSSC3, and WIF1 which lose function in osteosarcoma play a role in the autophagy.^63^, ^64^ For the development of drug resistance in osteosarcoma, some heat shock protein, such as HSP90AA1, is also related to autophagy.^65^, ^66^, ^67^ MicroRNA is also important in the formation of drug resistance of osteosarcoma, for example the miR‐143 can inhibit autophagy but the expression of miR‐143 decreases in osteosarcoma and expression of ATG2B and LC3‐II increases, which can promote autophagy to generate chemoresistance in tumors.^68^, ^69^, ^70^

### 2.4 Pyroptosis

Pyroptosis is mediated by pore‐forming proteins, which can form a pore on cell membrane and lead to the damage of cell membrane.^71^, ^72^, ^73^ It is kind of pro‐inflammatory cell death which is mediated by Caspase‐1. The activating Caspase‐1 can cleavage the pore‐forming proteins, and the cleavage pore‐forming protein can form membrane pore.^12^, ^74^ Hypoxia is an important characteristic for osteosarcoma.^74^ Hypoxia helps osteosarcoma cell evade the immune system and survive. In addition, the hypoxia can also induce the expression of hypoxia‐inducible factor (HIF), HMGB1, and toll‐like receptor 4, which can induce the metastasis of osteosarcoma. But the role of TLR4 is still unclear, asit may either promote or inhibit the metastasis.^17^, ^75^, ^76^, ^77^ In the presence of hypoxia, many of the signaling pathways or molecules involved in pyroptosis are activated thereby causing the generation of cellular pyroptosis.^78^, ^79^ For example, the damage to the mitochondrial structure reduces ROS production, which leads to cellular hypoxia and thus promotes cellular pyroptosis.^80^ In addition, the hypoxia and pyroptosis can enhance HMGB1 expression, which can activate ERK1/2 signaling pathway promoting tumor cell proliferation. Thus, in a sense, cellular pyroptosis can promote cancer progression.^81^ Experimental evidence that pyruvate dehydrogenase kinase 1 (PDK1) and lysyl oxidase (LOX) genes associated with hypoxia is associated with tumor metastasis and survival, while decorin (DCN) can inhibit tumor.^17^, ^79^, ^82^ What's more, the hypoxia will also inhibit the function of CD8+ T cell. CD8+ T cell contains magnanimous granzyme A, which can be sent to target cell to cause pyroptosis. So, inhibition of CD8+ T cell function can inhibit pyroptosis.^73^, ^74^, ^83^, ^84^ Caspase‐1 inhibits cancer cell migration,^85^ and GSDMA can regulate the pyroptosis to inhibit cancer cell development.^86^ Long noncoding RNA (lncRNA) influences on cellular pathways of pyroptosis through regulation of gene expression and thus has influence on tumor. For example, FOXD2‐AS1 is a proto‐oncogene, which can induce cancer. In addition, the AC090559.1 contributes to CASP1, GSDMA expression and thus promotes pyroptosis to inhibit osteosarcoma development.^87^ In addition, microRNAs have also been associated with osteosarcoma cell pyroptosis. For example, miRNA‐181a can inhibit NLRP3‐dependent cellular pyroptosis, so low expression of miRNA‐181a contributes to the occurrence of cellular pyroptosis and thus suppresses tumor growth.^88^ Many chemotherapeutic and non‐chemotherapeutic drugs can also be used to fight cancer by inducing cellular pyroptosis, such as decitabine and azacitidine induced GSDME to promote the production of cellular pyroptosis to treat cancer.^89^, ^90^ In addition to the chemotherapeutic drugs like decitabine and azacitidine, the non‐chemotherapeutic drug dioscin induces the formation of pore on the cell membrane of osteosarcoma cells and, thereby promoting apoptosis in osteosarcoma treatment.^91^ However, overproduction of pyroptosis can be detrimental to the body. Excessive cellular pyroptosis can lead to overproduction of pro‐inflammatory factors, which can cause tissue damage.^91^

### 2.5 Necroptosis

Necroptosis is a type of PCD that relies on three core proteins, receptor‐interacting protein kinase 1(RIPK1), receptor‐interacting protein 3 (RIP3), and mixed lineage kinase‐like protein (MLKL), to cause organelle swelling and alter the permeability of the cell membrane.^92^ Like other PCDs, necrotic apoptosis has a crucial role in the development of osteosarcoma. RIP1 plays a crucial role in necroptosis, TRA/GO reduces cIAP and Caspase‐8, the reduction of cIAP contributes to the formation of intracellular platform complexes, whereas the decrease in Caspase‐8 activity facilitates the formation of RIP1/RIP3/MLKL complexes, which promotes necroptosis and kills osteosarcoma cells.^93^, ^94^, ^95^, ^96^ Like pyroptosis, necroptosis‐associated lncRNAs and miRNAs are importantly linked to the development of osteosarcoma. lncRNAs can regulate target mRNAs, thereby promoting necroptosis in cancer cells.^97^ Some necroptosis‐associated lncRNAs are also associated with cancer. Necrotic apoptosis‐related lncRNAs can also affect OS by influencing immunity.^92^ Shikonin (SHK) can promote the RIP1 and RIP3‐dependent necroptosis to treat osteosarcoma.^98^, ^99^ As one of the most common kinds of necroptosis, the TNF‐mediated necroptosis is induced by the binding between TNF‐α and cell membrane, which can activate the downstream signal pathway leading to the formation of membrane complex and this can lead to necroptosis.^100^, ^101^ Some studies have shown that the herbs extraction emodin can treat cancer by activating TNF‐a induced necroptosis.^102^ Some NRlncRNA, such as AL391121.1, can influence the function of CD40 which is important cancer immunity.^103^ In the immune response to osteosarcoma, some immune cell such as macrophage can stimulate the three core necroptosis proteins to induce necroptosis, which is beneficial for cancer treatment.^104^, ^105^ For immune checkpoint, it is true that many immune checkpoints can protect tumor cell from immune system. Some studies showed that in low‐risk group of NRlncRNA, the immune checkpoint expression level is high.^103^

### 2.6 Cuproptosis

Cuproptosis, as a novel mode of programmed cell death, also has a very important role in the generation and development of osteosarcoma. A hallmark of copper death is that excess copper can interact with lipoylated proteins to affect the TCA cycle leading to cell death.^106^ Copper death has an important role in tumors such as melanoma, clear cell renal cell carcinoma, and hepatocellular carcinoma.^107^, ^108^, ^109^ Experimental evidence suggests that mitochondria‐dependent malignancies can be treated by copper death. Although there are fewer studies related to cuproptosis and OS, it has been experimentally demonstrated that cuproptosis can promote cancer generation by promoting certain genes that induce cancer production, for example, TGF‐beta, Wnt/beta‐catenin, and p53 signaling.^110^ Research has shown that the NGX6 can inhibit the Wnt/nt/wn that the NGX6 can to inhibit cancer cell metastasis. It has been demonstrated that high‐risk cuproptosis‐related Lncs (CRLncs) can downregulate immune cell function, which increases the risk of osteosarcoma, while low‐risk CRLncs decrease the risk of osteosarcoma.^111^ Different cuproptosis‐related genes (CRGs) have different effects on osteosarcoma, for example, FDX1 expression contributes to tumor cell survival and drug resistance, and FDX1 can also promote the osteosarcoma cell metastasis but not related to the cancer cell proliferation and the overexpression of FDX1 can also inhibit the tumor immune microenvironment (TIM).^110^, ^111^ TOMM20 contributes to colon cancer cell invasion suppressing the activity of immune cells such as CD8 T cells and CD4 T cells, aiding cancer cells in evading the immune system. Additionally, overexpression of TOMM20 can contribute to the development of drug resistance in tumor cells against immune‐based therapies, thus impacting tumor treatment.^112^, ^113^, ^114^ NDUFB9 expression contributes to the inhibition of cancer cell proliferation and metastasis. In the osteosarcoma tissue, FDX1, TOMM20, and NDUFB9 are all overexpress to promote the development of osteosarcoma. Besides these few genes associated with poor prognosis, there are also some genes associated with better prognosis but also bad, such as MFN2 and ATP6V1E1. The expression level of MFN2 is increased in osteosarcoma, and it can inhibit the immune system in osteosarcoma TIM. The level of ATP6V1E1 mRNA is downregulated in osteosarcoma, and the ATP6V1E1 mRNA expression dysregulation leads to immune imbalance in osteosarcoma.^115^, ^116^

## 3 MODULATION OF CELL DEATH IN OSTEOSARCOMA

With the development of molecular biology, we have discovered that various signaling pathways are intimately involved in cell development, particularly in relation to programmed cell death in cancer cells. Therefore, we have summarized several common signaling pathways.

### 3.1 PI3K/Akt signal pathway

Many experiments have shown that the alteration of the function of PI3K/AKT signal pathway is related to the proliferation, metastasis, and metabolism of osteosarcoma. The PI3K/Akt pathway is downstream of RTKs and G protein‐coupled receptors (GPCRs). PI3K is heterodimeric and consists of a catalytic subunit p110 and a regulatory subunit p85.

**FIGURE 2:**
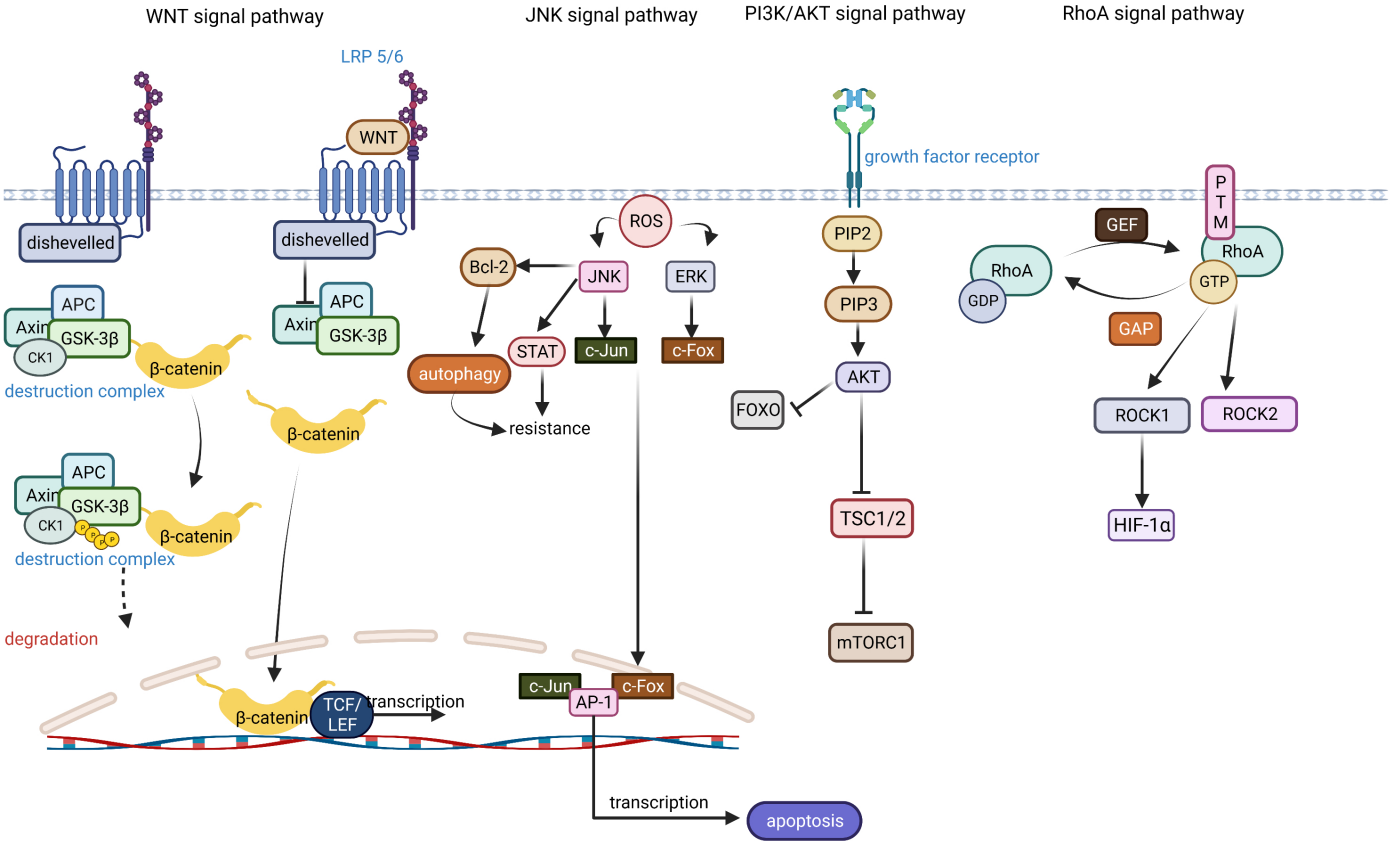
Core signal pathway in osteosarcoma. RhoA signal pathway: RhoA‐GDP complex binds to GTP in response to GEF and is immobilized at the cell membrane by PTM, and the GTP‐bound RhoA complex is active in promoting ROCK1/2 production and controlling cell death. PI3K/AKT signal pathway: The growth factor receptor contributes to the conversion of PIP1 to PIP3, which activates AKT, which inhibits FOXO and TSC1/2. Inhibition of FOXO reduces apoptosis, and inhibition of TSC1/2 contributes to the deregulation of the inhibitory effect of TSC1/2 on mTORC thereby promoting the action of motor, which inhibits autophagy production. JNK signal pathway: ROS can activate JNK and ERK to promote c‐Jun and c‐Fox binding to AP‐1 to regulate gene transcription. At the same time, JNK can activate STAT and Bcl‐2 to increase the viability of cancer cells and contribute to the development of chemoresistance. Wnt signal pathway: In the off state of wnt pathway, axin, APC, GSK3β, and CK1 form a destruction complex that phosphorylates β‐catenin and prevents it from entering the nucleus to participate in transcription. When wnt binds to the receptor Fzd, it hydrolyzes axin and protects β‐catenin from degradation, so that it can enter the nucleus to participate in transcription. This figure has been created at https://app.biorender.com (accessed on July 16, 2023).

According to the structure and function of phosphatidylinositide 3‐kinases (PI3K), it can be divided into three subclasses, namely classes I, II, and III, among which class I is the most widely studied.^117^ The activated PI3K can phosphorylate PIP2 to become PIP3, which acts as a second messenger and continues to phosphorylate downstream target proteins PDK1 and serine/threonine(AKT), leading to the activation of AKT. Activated AKT has diverse effects on a variety of downstream substrates, such as inhibition of FOXO, promotion of DNA repair, and inhibition of DNA replication to inhibit cancer. The key downstream substrate of AKT is the tuberous sclerosis complex (TSC), consisting of TSC1, TSC2, and TBC1D7; mammalian target of rapamycin (mTOR) has two forms, mTORC1 and mTORC2. The principle is that TSC2 can hydrolyze GTP in RHEB protein to convert to GDP and thus inactivate RHEB protein, which function is to activate MTORC1. However, AKT can phosphorylate TSC2, inhibiting the activity of TSC2 and activating mammalian target of rapamycin (mTOR) pathway.^118^ MTOR has two complex forms, namely MTORC1 and MTORC2. mTOR can be activated by AKT in many conditions, such the increase of AMP/ATP ratio which will cause energy deficit, anoxia, and TSC increase. When acidic environment is enhanced, the synthesis of mTORC1 can be promoted. The PIK3CA gene, part of the PI3K family, encodes the p110a catalytic subunit. PIK3CA mutations, common in Class I, disrupt signaling pathways, contributing to osteosarcoma. The SNP rs7646409 in PIK3CA intron triggers p110a overexpression, activating Akt signaling, suppressing osteosarcoma apoptosis, and promoting proliferation.^119^ About autophagy in osteosarcoma, the function of PI3K/Akt/mTOR signal pathway is complex. mTORC1 inhibits autophagy by phosphorylating ULK1 and Atg13 and weakening their binding.^120^ mTORC1 also phosphorylates ATG14L, inhibiting lipid kinase activity and providing an alternative autophagy inhibition pathway. Transcription factor EB (TFEB) regulates autophagy gene expression. mTORC1 can inactivate TFEB transcriptional regulation by phosphorylating Ser142 and Ser211 of TFEB. The involvement of mTORC2 in autophagy regulation is not fully understood. However, it has been suggested that mTORC2 might play a role in activating mTORC1 to modulate autophagy.^121^ Some studies have found that there is upregulation of PI3K/Akt and downregulation of PTEN in osteosarcoma (OS). PTEN acts as an upstream inhibitory signal of PI3K. Experimental evidence shows that promoting PTEN expression with honokiol can reduce excessive autophagy in osteosarcoma cells. Additionally, MiR‐1224–5p can inhibit PLK1 expression, thereby suppressing the PI3K/Akt/mTOR signaling pathway and inducing autophagy. Ginsenoside can also inhibit the PI3K/Akt/mTOR signaling pathway, induce cell autophagy, and apoptosis, thereby suppressing the development of osteosarcoma.^122^ The PI3K signaling pathway can be also activated by nutrient and growth factor, thereby inhibiting cellular autophagy, and promoting the proliferation and growth of osteosarcoma cells. However, cellular autophagy has a dual role in cancer development, with excessive autophagy promoting cancer cell death. In MG‐63 cells, a human osteosarcoma cell line, honokiol promotes the expression of the LC3 protein, thus inducing autophagy in osteosarcoma cells and leading to their death.^123^ Furthermore, research has shown that honokiol and diallyl disulfide (DADs) can also promote cancer cell autophagy by inhibiting the p53/PI3K/Akt/mTOR pathway, thereby achieving therapeutic effects.^124^ In addition, the DADs can also upregulated the Caspase‐3 and Bax, which can induce cell apoptosis and kill osteosarcoma.^125^ Wei et al. found that Rab‐interacting lysosomal protein (RILP) overexpressed in OS 143B and U2OS cells phosphorylates growth factor receptor binding protein‐10 Grb10 promotes autophagy by inhibiting PI3K/AKT/mTOR pathway^126^ (Figure 2).

### 3.2 WNT/β‐catenin signal pathway

WNT genes are found to be in most animals and play a considerable role in regulating cell homeostasis and tumorigenesis.^105^ The complete WNT signaling pathway is divided into two parts: upstream signaling and downstream signaling. Upstream signals mainly refer to WNT proteins as ligands to activate disheveled proteins (Dsh or Dvl) of target cells. The role of PORCN is to interact WNT and Wntless (WLS) molecules to produce palmitoylation reactions. Subsequently, WLS transport palmitoylated WNT proteins to the plasma membrane for secretion. In addition, the dysregulation of WNT/β‐catenin leads to proliferation and metastasis of osteosarcoma.^127^ It is the β‐catenin‐dependent signaling pathway that mainly causes osteosarcoma. The key to the open or closed state of the canonical pathway is the high or low level of β‐catenin protein expression.^128^ β‐catenin has a core of 12 repeat protein segments, which bind to enhance cell–cell adhesion junctions, consisting of C‐terminal and N‐terminal domains. The C‐terminus facilitates β‐catenin mediated transcription, while its the N‐terminus undergoes phosphorylation for degradation of β‐catenin. In the absence of WNT ligands, the pathway remains inactive. The key component is the scaffold protein axin. Adenomatous polyposis coli protein (APC) interacts with glycogen synthase kinase‐3 (GSK‐3) and casein kinase 1 (CK1) to form the β‐catenin destruction complex. Binding of the destruction complex to the N‐terminal domain of β‐catenin leads to its phosphorylation and subsequent degradation leading to the suppression of the WNT signaling pathway.^108^ In many study about osteosarcoma found that the expression level of axin in WNT/β‐catenin pathway are downregulate. The deletion of Axin 1 and disorder of APC can cause the WNT/β‐catenin signal pathway out of control and cause osteosarcoma development.^129^ The impact of β‐catenin on osteosarcoma development is complex, as evidenced by conflicting research findings. Some studies have shown low expression of β‐catenin in osteosarcoma, while others have demonstrated high expression. Therefore, we believe that the role of different WNT/β‐catenin pathways in osteosarcoma development depends on the specific downstream signaling factors and the particular subtype of osteosarcoma cells.^128^ KLF5 is a transcription factor that positively influences the WNT/β‐catenin signaling pathway. It enhances the expression of β‐catenin and interacts with it to promote its translocation into the nucleus. There, it binds to downstream transcription factors of the T‐cell factor/lymphocyte enhancer factor (TCF/LEF) family, leading to increased expression of cyclin D1. This process can be inhibited by ML264, which acts as a suppressor of KLF5. Studies have shown that ML264 exhibits significant therapeutic effects in osteosarcoma.^130^ WNT promotes the aggregation of β‐catenin, but the GSK‐3β/APC/Axin complex can also phosphorylate β‐catenin, thereby promoting its degradation. Therefore, GSK‐3β can inhibit the WNT/β‐catenin signaling pathway, leading to apoptosis and inhibition of proliferation in osteosarcoma cells.^131^ Additionally, downregulation of β‐catenin simultaneously inhibits the expression of Runx2, a pro‐apoptotic gene highly expressed in osteosarcoma cells. Heat shock protein 90 (HSP90) can reduce the expression of Runx2 by inhibiting the action of β‐catenin, thereby promoting apoptosis and inhibiting proliferation in osteosarcoma cells.^128^ Lnc‐MAP6‐1:3 inhibits β‐catenin expression along with its downstream targets TEF1, c‐Myc, and cyclin D, leading to reduced cellular proliferation and increased apoptosis.^132^ In addition to apoptosis, the WNT/β‐catenin pathway also plays a significant role in osteosarcoma autophagy regulation. Treatment with XAV939, a Wnt/β‐catenin signaling inhibitor, resulted in elevated expression of the autophagy‐related gene Beclin 1 in MG63 cells. This led to decreased gemcitabine‐induced apoptosis and enhanced drug resistance.^127^

### 3.3 STAT3 signal pathway

The signal transducer and activator of transcription 3 (STAT3) pathway plays a critical role in numerous biological processes, including embryonic development, immune response, and cell migration. Activation of this pathway is swiftly triggered by molecules such as IL‐6 and growth factors like EGF and PDGF. Upon activation, STAT3 forms dimers and binds to DNA promoters, thereby regulating the transcription of target genes. Among the STAT family members, STAT3 stands out as an oncogene, frequently activated in various cancer types, particularly osteosarcoma.^133^

Research indicates that both transducer and activator of transcription 3 (STAT3) and cellular‐Myc (c‐Myc) are highly expressed in the malignant tumors.^134^ Interleukin‐6 (IL‐6) binds to their receptors IL‐6 receptor, resulting in the release IL‐6 family cytokines receptor‐associated Janus kinases (JAKs). It leads to the phosphorylation and dimerization of STAT3. STAT3 is the proto‐oncogene and cellular targets participating in the development of the osteosarcoma.^133^ Upon activation, STAT3 translocases into the nucleus, where binds to consensus response elements in the promoters of target genes, regulating their transcription. These genes include those involved in cell proliferation and survival, such as c‐Myc, Bcl‐2, survivin, and cyclins. It will upregulate the expressions of these genes to promote the formation, proliferation, invasion, and transfer of human osteoma cells. The nuclear transcription factor c‐Myc is a nuclear phosphoro‐protein that plays a crucial role in the cell cycle, apoptosis, protein synthesis, and cell transformation. It is a protein closely associated with the development of cancer. In cell cycle, it can induce the activity of cyclin D‐CDK2 to accelerate the cell cycle from G1 to S phase. What's more, the other cell cycle related protein such as cyclin D1 and D2, cyclin E, cycle B1, and CDK4 can also infected by c‐Myc which can influence cell cycle. Besides, c‐Myc can also influence other signal pathway such as NF‐κB and HIF to influence cell cycle. In osteosarcoma tissue, overexpression of c‐Myc promotes cancer cell proliferation.^135^, ^136^ c‐Myc has a dual role in apoptosis. On one hand, it induces the expression of ARF, which binds to mouse double minute 2 (Mdm2) and increases the level of p53, leading to apoptosis and cancer cell death. However, dysregulation of c‐Myc can also inhibit apoptosis, thereby promoting osteosarcoma growth.^137^

Research indicates that targeting the STAT3 signaling pathway is a promising approach for osteosarcoma treatment, particularly in addressing programmed cell death and other forms of programmed cell death. Diosmetin is proved to be used to treat human osteosarcoma cells.^134^, ^138^, ^139^ Diosmetin can cause the cell cycle arrest that occur in the G2/M phase. In addition, the G2/M phase regulation point is the key point for signal transmission inside and outside the cell, integration, and collection to the nucleus to regulate cell proliferation in cell cycle regulation.^134^, ^140^, ^141^ The theory of treatment is that it can inhibit STAT3 phosphorylation and dimerization as well as reduce c‐Myc expression to influence the cell cycle. It will promote the apoptosis and inhibit proliferation of human osteosarcoma cells through inhibiting the STAT3/c‐Myc signaling pathway. Research has found that LRP8 have a high expression level in osteosarcoma and activates STAT3 which can promote the expression of PD‐L1 in osteosarcoma. However, PD‐L1 can inhibit T cells, preventing T cell‐induced apoptosis in osteosarcoma. Experimental evidence has shown that reducing the level of LRP8 can promote apoptosis in osteosarcoma and inhibit the unlimited proliferation of osteosarcoma cells.^142^ The sporoderm‐broken spores of *Ganoderma lucidum* (BSGWE) can also downregulate the phosphorylation of STAT3 to decrease the expression of PD‐L1, which can promote the apoptosis in osteosarcoma.^143^

### 3.4 RhoA signal pathway

The Ras homolog (Rho) family of small GTPase plays a crucial role in cell cycle, cell morphology, and cell migration.^144^ These proteins undergo conformational changes between active and inactive states depending on their binding to GTP/GDP. GTPase‐activating proteins (GAPs) facilitate the hydrolysis of GTP to GDP thereby inactivating Rho, while guanine nucleotide exchange factors (GEFs) promote the binding of GTP to activate Rho.^145^, ^146^ At the carboxyl terminus of human Rho, there are a post‐translational modification region (PTM), which can help Rho insert on the cell membrane to induce its signal pathway and get it function.^147^ Rho‐kinase (ROCK) is a kind of serine/threonine kinase, which is an important downstream factor of Rho.^148^ The ROCK have two main kinds one is ROCK1 which is important in the mitochondrial dynamics and cell differentiation, the other is ROCK2, which is important in cell death and inflammation.^149^

The RhoA‐ROCK‐LIMK2 signal pathway is important for the osteosarcoma migration and related to PCD. Programmed death ligand‐2 (PD‐L2) is an upstream signaling molecule of the RhoA signal pathway. It is known as a co‐stimulatory molecule that can bind to the PD‐1 receptor to inhibit the activity of T cells, thus playing an important role in tumor migration and progression. Because there is experimental evidence showing that in metastatic osteosarcoma, such as osteosarcoma metastasized to the lungs, the expression level of PD‐L2 is much higher than in non‐metastatic primary osteosarcoma.^59^ Experimental evidence has shown low expression of PD‐L2 can lead to decreased expression of beclin‐1, thereby inhibiting the RhoA‐ROCK‐LIMK2 pathway.^59^ This means that PD‐L2 can activate RhoA‐ROCK‐LIMK2 signal pathway. The dependence lies on the repulsive guidance molecule BMP co‐receptor B (RGMb). RGMb, acting as a secondary receptor for PD‐L2, forms a BMP‐BMPR‐RGMb‐neogenin (BBRN) super complex. Through this super complex, PD‐L2 regulates downstream signaling pathways such as RhoA‐ROCK‐LIMK2.^150^ Numerous studies have shown that PD‐L2 is expressed in many patients with osteosarcoma and that there is a strong association between PD‐L2 and migration and invasion of osteosarcoma.^59^ Autophagy exhibits a dual role in cancer cell migration and invasion—it can either promote or inhibit these processes. RhoA‐ROCK‐LIMK2 signaling pathway is associated with autophagy in tumor cells. It was demonstrated that PD‐L2 acted as an upstream factor of RhoA‐ROCK‐LIMK2 and inhibited the RhoA‐ROCK‐LIMK2 signaling pathway when PD‐L2 expression was downregulated, which helped to inhibit osteosarcoma cell autophagy and this can inhibit the metastasis of osteosarcoma. In addition, PD‐L2 can also induce autophagy through beclin‐1, as another upstream signal of the RhoA‐ROCK‐LIMK2 signaling pathway, beclin‐1 can also control cellular autophagy by regulating RhoA‐ROCK‐LIMK2 signaling pathway and also influence the tumor metastasis.^59^, ^151^, ^152^, ^153^

In addition to cellular autophagy, RhoA‐ROCK has a crucial role in apoptosis. The RhoA‐ROCK signaling pathway regulates control of integrin‐mediated interactions between cells and cellular matrix thereby regulating cell adhesion.^154^ TRIM59 stabilizes PDCD10, which also acts as an upstream signal of the Rho‐ROCK signaling pathway and inhibits it, thereby promoting metastasis and inhibiting apoptosis.^155^ Some experiments had shown that using of lipophilic statin drugs can induce apoptosis in osteosarcoma cells, thereby inhibiting the development of osteosarcoma. Statin drugs induce apoptosis in osteosarcoma cells by altering the localization of RhoA, causing its relocation from the cell membrane to the cytoplasm and leading to RhoA isoprenylation. This process triggers apoptosis. Furthermore, statin‐induced apoptosis is attributed to the inhibition of survival signals. By inhibiting RhoA‐GTPase activity, statin drugs block the p42/p44‐MAPKs survival pathway, accelerating apoptosis in osteosarcoma cells.^156^, ^157^

### 3.5 JNK signaling pathway

As one of the three well‐known mitogen‐activated protein kinase (MAPK), the c‐Jun N‐terminal kinase (JNK) signal pathway is important for the converting extracellular stimuli into intracellular signals.^158^, ^159^ The JNK kinase family have JNK1, JNK2, and JNK3, and the JNK1 and 2 are the most important JNK kinase family. The upstream molecules of JNK protein mainly include growth factors and pro‐inflammatory cytokines, such as TNF‐a and IL‐1, which can activate JNK protein. Additionally, certain environmental factors such as UV radiation and toxins can also exogenously activate JNK protein.^160^ In addition, JNK 1 increase the transcription factor function by phosphorylating the composition of activator protein‐1 (AP‐1), such as c‐Jun, ATF2, and c‐Myc.^161^, ^162^

The JNK signaling pathway, as a factor closely related to cell survival, growth, and value‐added, is also closely related to tumor development. The JNK signaling pathway can affect tumor microenvironment generation by regulating TGF‐β to promote tumor development.^163^, ^164^ JNK can also play a role in immune escape from and tumor cell through the JNK‐STAT3 pathway.^165^ It can protect the cancer cell from apoptosis and increase the chemoresistance by decreasing the ROS level, which can prevent tumor from death.^166^ What's more, it can promote cancer cell proliferation by regulating the expression level of miRNA‐92a and glucose‐regulated protein 78 (GRP78). It can also upregulate the expression of early growth response gene‐1 (Egr‐1) to increase cancer cell proliferation.^167^ The JNK can inhibit apoptosis by activating autophagy.^168^ The two main JNK kinase family members JNK1 and JNK2 have different role in cell survival. JNK1 can help cancer survival while JNK2 can cause cell death through apoptosis.^165^ JNK1 promotes TBP production and thus tumor development and inhibits apoptosis through JNK1/STAT3 signaling.^169^ But in contrast, JNK1 can mediate TNF‐α‐induced apoptosis.^170^ For autophagy, JNK regulates autophagy by affecting autophagy‐related genes and induces autophagy through Bcl‐2 phosphorylation, which contributes to cancer cell survival.^171^

In the development of osteosarcoma, activated JNK protein, together with c‐Jun, c‐FOS, acts on core‐binding factor 1, leading to the transformation of osteoblasts into cancer cells. Therefore, dysregulation of the JNK signaling pathway may be a key factor in the cause of osteosarcoma. Additionally, activation of MMP family proteins, downstream proteins of JNK, can promote the migration of osteosarcoma cells.^172^, ^173^ But under some situation, the different way to activate JNK/c‐Jun signal pathway can also promote the apoptosis and autophagy in osteosarcoma. Therefore, targeting the JNK signaling pathway can be used to intervene in the pathogenesis and progression of osteosarcoma for therapeutic purposes. Experimental evidence has shown that the JNK inhibitor SP600125 significantly reduces the transcriptional impact of AP‐1, thereby decreasing the migration of osteosarcoma cells^163^ Metformin increases the ROS‐mediated JNK/c‐Jun signal pathway to induce apoptosis in osteosarcoma. The accumulation of ROS induces a JNK/c‐Tun cascade reaction, which leads to the generation of cell apoptosis and autophagy. Therefore, metformin induces a significant ROS production, thus exerting a resistance effect against osteosarcoma.^174^ The curcuminoid HO‐3867 can activate apoptosis in osteosarcoma through JNK signal pathway.^175^ A small molecule TGF‐βRI/ALK5 inhibitor (RepSox) promotes apoptosis to inhibit osteosarcoma cell proliferation, and it acts by inhibiting TGF‐βR and thus affecting the JNK and Smad3 signaling pathway.^176^ As the flavonoid monomer, cardamonin (CAR) can stimulate JNK signal pathway which can phosphorylate BH3 protein or use P53 signal pathway to promote cell apoptosis to inhibit osteosarcoma development.^177^ As a kind of flavanol, fisetin can increase the expression of ZAK which can activate JNK/ERK signal pathway by promoting the c‐Jun and c‐Fos binding to AP‐1 to cause apoptosis of osteosarcoma cell.^178^ The c‐Jun and ‐Fos are the downstream factor of JNK and ERK. The IRE1‐ASK1‐JNK is also essential for the osteosarcoma development. The production from bacillus subtilis named surfactin can cause stress on ER, which can activate IRE1‐ASK1‐JNK signal pathway and lead to apoptosis of osteosarcoma cell.^179^

## 4 NEW TREATMENTS ASSOCIATED WITH PROGRAMMED CELL DEATH

The standard treatment approach for localized osteosarcoma involves a chemotherapy regimen called MAP, comprising high‐dose methotrexate, doxorubicin, and cisplatin, administered before and after surgical resection. In cases of relapsed or metastatic osteosarcoma, metastasectomy surgery may enhance patient survival outcomes. For osteosarcoma that are unresectable or exhibit high recurrence rates, chemotherapy with gemcitabine and docetaxel or etoposide and ifosfamide is often considered, although survival rates remain modest.^180^ Currently, post‐chemotherapy surgical resection remains an integral part of osteosarcoma treatment, typically utilizing wide margin surgery, with a surgical failure rate of less than 5% post‐chemotherapy.^181^ Surgical resection is classified into three levels: R0 to R2. R0 involves complete removal of cancer cells with no residual tumor at the margins, known as negative margins. R1–R2 indicate varying degrees of residual tumor. Studies have shown that for surface osteosarcoma, both R0 and R1 resections are effective in preventing recurrence by ensuring negative margins. However, there is not a precise standard for negative margins. Research suggests that by carefully reducing the distance between resection and tumor margins, limb function can be preserved while maintaining a low recurrence risk. They found that when the negative margin is less than 1 mm or precisely at the margin under a microscope, it is acceptable.^182^

Although past treatment methods have shown relatively good results, new treatment methods for osteosarcoma, especially those targeting programmed cell death, need to be developed to improve prognosis and minimize patient burden. As an important regulatory mechanism to modulate the development of osteosarcoma, PCD could be a novel target for the treatment of osteosarcoma, thereby reducing its aggressiveness and migration. GSK343, a methionine inhibitor, was shown to induce apoptosis by expressing cleaved Caspase‐3 and poly ADP‐ribose polymerase (PARP) in a human osteosarcoma cell line: Saos2 cell line, which can kill osteosarcoma cell.^183^ For cellular autophagy, GSK343 inhibits P62 expression and promotes LC3‐II production thereby promoting cellular autophagy, and excessive cellular autophagy can kill tumor cells.^183^ GSK343 inhibits the expression of enhancer of zeste homolog 2 (EZH2), a polycomb group proteins (PcG), in osteosarcoma cells and EZH2 is highly expressed in osteosarcoma cells. EZH2 is a novel target for effective osteosarcoma treatment.^184^, ^185^ In the JNK signal pathway, RepSox can regulate the TGF‐βR function to influence the JNK signal pathway to induce osteosarcoma cell apoptosis and CAR can help JNK signal pathway phosphorylate BH3 protein to induce apoptosis.^176^, ^177^ What's more, the surfactin can induce apoptosis through IRE1‐ASK1‐JNK signal pathway.^179^ The metformin can induce ROS/JNK mediated apoptosis of osteosarcoma cell.^174^ The Rho signaling pathway, which influences cell migration, adhesion, and proliferation, represents a significant therapeutic target for curtailing osteosarcoma cell migration.^186^ Zoledronic acid has the capability to impede the prenylation of Rho, which is crucial for Rho's transmission of extracellular signals, by inhibiting geranylgeranyl diphosphate (GGPP) and farnesyl diphosphate (FPP).^187^ The zoledronic acid have been proved to play an important role in inhibition of osteosarcoma cell migration to lung.^188^ As PD‐L1/2 is upregulated in osteosarcoma, PD‐L1/2 can bind to PD‐1 and thus inhibit the PCD of osteosarcoma cells. Clinical studies have demonstrated that pembrolizumab prevents PD‐L1/2 from binding to PD‐1 and thus promotes the PCD of osteosarcoma cells, thus achieving therapeutic effects.^189^ As a reactive oxygen species‐dependent PCD, the iron death‐associated signaling pathway may also serve as a therapeutic target for osteosarcoma. The bavachin can increase expression of transferrin receptor such as DMT1 or P53 and inhibit the secretion of STAT3. P53 can inhibit SLC7A11, so, the bavachin can inhibit STAT3/P53/SLC7A11 axis to induce ferroptosis in osteosarcoma cell.^52^ JAK2/STAT3 signal pathway is an important for cellular apoptosis, migration, and proliferation, and it can be a new target on the treatment of osteosarcoma.^190^ As a natural isoquinoline alkaloid, liensinine can cause ROS accumulation to inhibit JAK2/STAT3 signal pathway, which can help cell proliferation and inhibit apoptosis. The liensinine can also increase the expression of Bax, Caspase‐3, and RARP, and decrease the expression of Bcl‐2 and cyclin D1. This can also induce the apoptosis in osteosarcoma cell.^190^ Pterostilbene can downregulate JAK2/STAT3 signal pathway to induce apoptosis of osteosarcoma cell. As a protein signaling pathway that affects cellular proliferation, differentiation, apoptosis, tumor microenvironment angiogenesis, and tumor cell metastasis, STAT3/c‐Myc is also critical in the treatment of osteosarcoma.^134^ For example, diosmetin and toosendanin can inhibit phosphorylation and dimerization of STAT3, which can inhibit the expression of c‐Myc. The decrease in c‐Myc can inhibit osteosarcoma cell proliferation and increase apoptosis.^134^ Cepharanthine (CEP) can inhibit some gene such as c‐Myc and Bcl‐xL which are the target gene of STAT3 to play its antitumor function^191^ (Table 1).

**TABLE 1:** The potential therapy methods in osteosarcoma.

Therapy method	Typical molecule or drug	Target site	Mechanism	Disadvantage	Advantage	References
Inhibition of EZH2 mediated by GSK343	GSK343	Casp‐3, PARP, P62, LCH‐II	1. It can induce casp‐3 and PARP expression to induce apoptosis. 2. It can inhibit p62 expression and promote LC3‐II production to induce autophagy	Role in solid tumors is unclear. Lack of clinical trials	Significantly promotes death in human osteosarcoma	183, 192
Therapy on JNK signal pathway	Surfactin, metformin, RepSox, and CAR	Surfactin: IRE1‐ASK1‐JNK Metformin: ROS/JNK RepSox: TGF‐βR CAR: BH3 protein	They can induce apoptosis through different JNK signal pathway.	Lack of clinical trials Mechanism of growth inhibition of human osteosarcoma is unclear	Strong inhibitory effect on osteosarcoma cell proliferation	174, 176, 179, 193
Therapy on Rho signal pathway	Zoledronic acid	FPP and GGPP	It can inhibit the Rho signal pathway through inhibition of FPP and GGPP	Lack of clinical trials and only have experiments performed in osteosarcoma cell lines.	Effectively inhibit migration of osteosarcoma to the lungs	^194^
Immunotherapy	Pembrolizumab	PD‐1 and PD‐L1/PD‐L2 binding	It can inhibit binding of PD‐1 and PD‐L1/PD‐L2 binding, which can inhibit PCD of OS cell	The detail mechanism is unclear. Have some side effects such as nausea, anemia, and anorexia	Clinical studies are available to demonstrate feasibility	
STAT3/P53/SLC7A11	Bavachin	Transferrin receptor, STAT	It can increase p53 secretion and decrease STAT3 secretion to inhibit STAT3/p53/SLC7A11 axis to cause ferroptosis in OS cell	P53 have a dual role in ferroptosis, and its mechanism is unclear.	Has been shown to have good anticancer properties	^52^
JAK2/STAT3 signal pathway	Liensinine, Pterostilbene	JAK2/STAT3	They can inhibit JAK2/STAT3 signal pathway to induce apoptosis	The detailed mechanism of action is unknown	Less toxic Some experience in clinical use	^190^
STAT3/c‐Myc signal pathway	Diosmetin, toosendanin, Cepharanthine	c‐Myc	They can inhibit expression of c‐Myc in STAT3/c‐Myc to induce apoptosis in OS cell	Antitumor characteristics in osteosarcoma are unclear	Fewer adverse reactions	134, 191, 195

## 5 CONCLUSIONS AND OUTLOOK

PCD is an important mechanism for normal physiological activity of cells such as cellular proliferation, migration, growth, and differentiation. In this review, we introduce different roles of PCD in the osteosarcoma development and the function of different signal pathway. For example, the apoptosis and ferroptosis can cause the death of osteosarcoma cell, and some drug can block System XC‐ to induce ferroptosis to inhibit the development of osteosarcoma. The autophagy has a dual role in osteosarcoma. Autophagy can either protect or destroy osteosarcoma, protection or destruction depending on the environment. In osteosarcoma, hypoxia can lead to pyroptosis, which can kill osteosarcoma cells. For necroptosis in osteosarcoma, the formation of the RIP1/RIP3/MLKL complex can induce necroptosis in osteosarcoma cells. Cuproptosis and osteosarcoma are related but specific roles and mechanisms still need to be studied.

The different signal pathways regulate different types of PCD. The activation of mTOR can inhibit autophagy and promote cell proliferation, and the PI3K/AKT/mTOR signal pathway contributes to angiogenesis in the tumor microenvironment. The WNT signal pathway is transmitted by beta‐catenin. Beta‐catenin phosphorylates TSC and thus induces autophagy. As a molecule that regulates cellular activity, c‐Myc inhibits apoptosis in osteosarcoma cells, while the STAT3/c‐Myc signaling pathway contributes to the expansion and development of osteosarcoma cells. The RhoA‐ROCK‐LIMK2 signaling pathway contributes to osteosarcoma cell migration and is implicated in cellular autophagy and apoptosis. The JNK signal pathway can decrease the level of ROS, which can protect the osteosarcoma cells from apoptosis, and the JNK pathway can promote osteosarcoma cell proliferation.

To sum up, since different PCDs and their associated signaling pathways are critically linked to the growth and death of osteosarcoma cells, a variety of drugs targeting PCD, and its signal pathway may be used to treat osteosarcoma in the future. For example, there are experiments on cuproptosis that have found that in a prognostic model of osteosarcoma, four drugs, AUY922, bortezomib, lenalidomide, and Z.lle.CHO, can inhibit the development of osteosarcoma.^111^ In addition, diosmetin have been shown that it can be used to treat human osteosarcoma through STAT3/c‐Myc signal pathway. Bavachin can change the morphology of mitochondria to induce the ferroptosis in cancer cell which can treat osteosarcoma. Cisplatin is a common chemotherapeutic agent for osteosarcoma treatment but tends to inhibit iron death leading to resistance. So, promoting iron death could be a new approach to counteract tumor resistance to cisplatin. In summary, many of the signal pathway related to osteosarcoma can be the target of treatment of osteosarcoma. However, there are still many challenges to achieve this, but we believe that in‐depth experimental and clinical research will further promote the development of PCD as an important target for the treatment of osteosarcoma in the future.

## AUTHOR CONTRIBUTIONS

**Suqing Liu:** Visualization (equal); writing – original draft (equal). **Chengtao Liu:** Visualization (equal); writing – original draft (equal). **Yian Wang:** Validation (equal); visualization (equal). **Jiewen Chen:** Formal analysis (equal); methodology (equal). **Yujin He:** Data curation (equal). **Kaibo Hu:** Investigation (equal). **Ting Li:** Project administration (equal). **Junmei Yang:** Writing – review and editing (equal). **Jie Peng:** Conceptualization (equal); data curation (equal); project administration (equal); supervision (equal); validation (equal); writing – review and editing (equal). **Liang Hao:** Data curation (equal); project administration (equal); supervision (equal); writing – review and editing (equal).

## FUNDING INFORMATION

This study was supported by the Distinguished Youth Fund Project of Jiangxi Natural Science Foundation (Grant number [20212ACB216011]), the Key Project of the Science and Technology Research Program of the Jiangxi Provincial Department of Education (Grant numbers [GJJ190014]), the National College Students' Innovative Entrepreneurial Training Plan Program (Grant numbers [202110403003] and [202210403001]), and the Innovation and Entrepreneurship Training Program for College Students in Jiangxi Province (Grant number [S202210403002] and [S202310403034]).

## CONFLICT OF INTEREST STATEMENT

The authors have no competing interests to declare that are relevant to the content of this article.

## Data Availability

Data sharing is not applicable to this article as no datasets were generated or analyzed during the current study.
